# Temporo mandibular joint ankylosis

**DOI:** 10.1016/S1808-8694(15)30748-5

**Published:** 2015-10-19

**Authors:** Belmiro Cavalcanti do Egito Vasconcelos, Gabriela Granja Porto, Ricardo Viana Bessa-Nogueira

**Affiliations:** 1 Doctor, coordinator of the dentistry octoral program – buccomaxillofacial surgery and traumatology unit, UPE; 2 Specialist in buccomaxillofacial surgery and traumatology, Pernambuco University; graduate student in the dentistry master's degree course on buccomaxillofacial surgery and traumatology, Pernambuco University; 3 Master in buccomaxillofacial surgery and traumatology, Pernambuco University; graduate student in the dentistry doctoral degree course on buccomaxillofacial surgery and traumatology, Pernambuco University. Dentistry School, Pernambuco University (FOP-UPE)

**Keywords:** ankylosis, temporomandibular joint, surgery, literature review

## Abstract

Ankylosis may be defined as joint surfaces fusion. The treatment of temporomandibular joint ankylosis poses a significant challenge because of the high recurrence rate.

**Aim:**

The aim of this study is to report six cases treated by joint reconstruction, evaluate the results of these surgeries and review the literature.

**Methods:**

The sample in this retrospective study was obtained from the records of the university hospital, patients who had to undergo ankylosis treatment by alloplastic or autogenous graft between March 2001 and October 2005. Pre- and post-operative assessment included a throughout history and physical examination to determine the cause of ankylosis, the Maximum mouth opening (MMO), etiology and type of ankylosis, recurrence rate and presence of facial nerve paralysis.

**Results:**

The mean MMO in the pre-operative period was 9.6 mm (0 mm to 17 mm) and in the post-operative period it was of 31.33 mm (14 mm to 41 mm), there was no facial nerve paralysis and there was recurrence in just one case.

**Conclusion:**

The joint reconstruction with alloplastic or autogenous grafts for the ankylosis treatment proved to be efficient in relation to the post-operative MMO, recurrence and joint function.

## INTRODUCTION

Ankylosis may be defined as the fusion of joint surfaces by bone or fibrous tissue.^1^ Temporomandibular joint (TMJ) ankylosis is a condition that may cause chewing, digestion, speech, esthetic, hygienic and psychological disorders.^2^, ^3^, ^4^

TMJ ankylosis may be classified according to the site (intra or extra-articular), type of tissue involved (bony, fibrous or fibro-osseous tissue) and the degree of fusion (complete or incomplete).^4^, ^5^, ^6^ According to Sawhney, it may also be classified into type I, in which the condyle is present and there are only fibrous adhesions; type II, in which there is bone fusion, the condyle is remodeled, and the medial pole is intact; type III, in which there is an ankylotic block, the mandibular ramus is fused to the zygomatic arch, the medial pole remains intact; and type IV, in which there is true ankylotic block and the anatomy is deranged because the ramus is fused to the skull base.^7^

Various factors may cause TMJ ankylosis, such as trauma, local and systemic inflammatory conditions, neoplasms, and TMJ infection.^3^^,^^5^ The most common etiological factors are trauma and infection.^3^ Su-Gwan^3^ studied seven operated patients and found that trauma was the main cause of ankylosis (85.7%). Roychoudhury et al.^2^ studied 50 patients and found that trauma was the cause of ankylosis in 86% of these cases.

A number of techniques have been described for the treatment of this condition in the literature. These include simple arthroplasty^8^, interposition arthroplasty^3^ and joint reconstruction using alloplastic or autogenous materials.^5^^,^^9^ This paper aimed to describe six clinical cases treated by the joint reconstruction technique using autogenous or alloplastic grafts, to assess the outcomes, and to review the literature on this theme.

## MATERIAL AND METHOD

A cross-sectional historical cohort study was undertaken between March 2001 and October 2005 in the city of Recife, Pernambuco state. The Pernambuco University Research Ethics Committee approved the study (number 099/06). The sample population was obtained from the patient files of the Oswaldo Cruz University Hospital (HUOC-UPE); patients had to have undergone surgery for the treatment of ankylosis by alloplastic or autogenous reconstruction.

Files were consulted for pre-, intra-, and immediate postoperative data. Patients were invited to a return visit for data verification and for late postoperative follow-up. Data was collected on the maximum mouth opening, the etiology, the type of ankylosis, the treatment, recurrences, and facial nerve injuries.

The type of ankylosis was classified according to Sawhney's classification into types I, II, III and IV apud Schobel et al.^7^ Facial nerve injury, if present, was noted and monitored by comparing pictures taken at various pre- and early/late postoperative follow-up dates.

Patients aged 16 years or above were considered as adults; patients aged below 16 years were considered as children.

## RESULTS

Table 1 shows data on the number of patients, type of treatment, age, sex, etiology, type of ankylosis, joints involved, recurrences and facial nerve injury.

**Table 1:** Epidemiological data on the operated cases.

No	Age	Sex	Etiology	Involvement	Type of ankylosis	Type of graft	Recurrence	Nerve injury
1	8	F	Cong.	Bilateral	IV	Autogen.	YES	NÕ
2	16	M	Infection	Unilateral	II	Autogen.	NO	NO
3	22	M	Trauma	Unilateral	III	Autogen.	NO	NO
4	17	F	Infection	Unilateral	III	Autogen.	NO	NO
5	20	M	Cong.	Unilateral	III	Autogen.	NO	NO
6	22	M	Infection	Unilateral	III	Autogen.	NO	NO

The mean follow-up period was 29.16 months (from 9 months to 56 months). The mean preoperative maximum mouth opening was 9.6mm (0mm to 17mm) and the mean postoperative maximum mouth opening was 31.33mm (14mm to 41mm) (Table 2).

**Table 2:** Pre- and postoperative measurements of mouth opening.

		Maximum mouth opening	Maximum mouth opening
No	Follow-up	Preop.	Postop.
1	56 months	0mm	14mm
2	16 months	10mm	29mm
3	26 months	15mm	35mm
4	36 months	8mm	41mm
5	32 months	17mm	31mm
6	9 months	8mm	38mm

**Figure 1:**
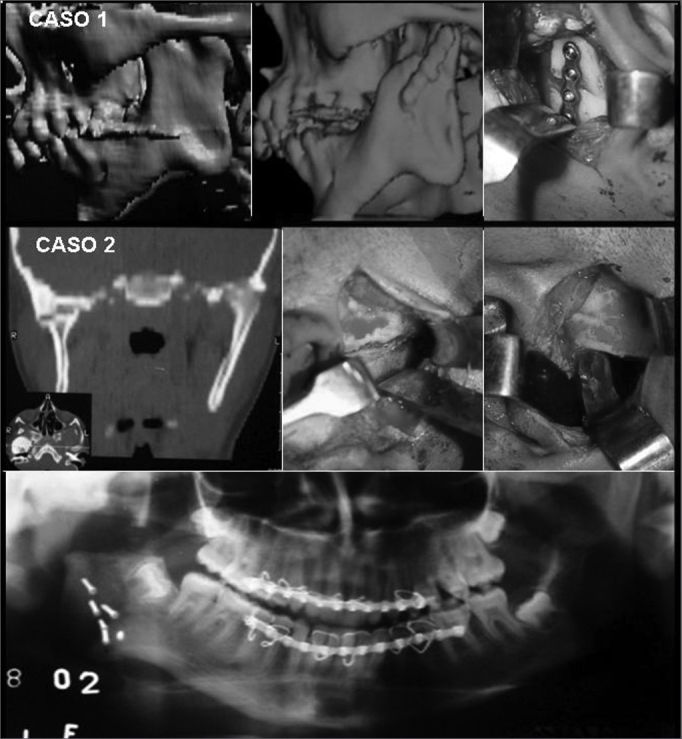
Case 1: Pre-, intra-, and postoperative radiological findings. Case 2: Pre-, intra-, and postoperative radiological findings.

Ellis and Zide's10 preauricular approach for ankylosis was used in all of the cases, under general anesthesia. The osseous or fibrous block was removed with troncoconical drills (703) and chisels until attaining mandibular movement. Next, the glenoid fossa was reconstructed, if necessary. Ipsilateral coronoidectomy was done in all of the cases; in autogenous reconstruction cases, the coronoid process was used for reconstructing the condyle (Figure 1). Contralateral coronoidectomy was done only if intraoperatory maximum mouth opening (35mm) was not attained. In allogenous reconstruction cases, an acrylic resin condyle anatomy prosthesis was placed (Figure 1).

Both grafts were fixed by an internal rigid fixation system using 2.0mm miniplates. In all of the patients, vacuum drainage tube was placed at the end of the surgery and kept in place for 48 hours.

All of the patients were referred to a physical therapist for monitoring, 15 days after surgery.

## DISCUSSION

Surgery of TMJ ankylosis does not yield fully predictable results. The type of ankylosis and the patient's age should be evaluated when planning for surgery. An assessment of the type of ankylosis should define its site as intra- or extra-articular, unilateral or bilateral, and fibrous or osseous. Superior results are expected in unilateral fibrous ankylosis cases and in those with less bone involvement, compared to bilateral osseous ankylosis cases.

A number of treatments for this condition have been described in the literature, including simple arthroplasty,^8^^,^^11^ interposition arthroplasty^3^^,^^12^ – using temporal muscle fascia, ear cartilage or alloplastic material – and reconstruction of the joint using acrylic, titanium, or autogenous material prostheses.^5^^,^^9^ There is, however, no consensus in the literature about the best treatment in these cases; results have varied and recurrence rates are still high, which is a major problem when treating this condition.^5^ There was one recurrence in the current study, possible because the patient had bilateral type IV ankylosis and severe micrognathia that was not corrected simultaneously with the ankylosis. The patient's preoperative maximum mouth opening was 0mm and reached 14mm postoperatively.

Kaban et al.^13^ described a protocol for the treatment of TMJ ankylosis in 14 patients with a one-year follow-up. According to the paper, this protocol was ideal for treating this condition; it consists of: aggressive resection, ipsilateral coronoidectomy, contralateral coronoidectomy if needed, interposition with temporal fascia or cartilage, reconstruction of the ramus with a costochondral graft, rigid fixation, movement as soon as possible, and aggressive physical therapy. This protocol was applied to all of the sample patients in terms of the resection, coronoidectomy, graft reconstruction of the ramus, rigid fixation and aggressive physical therapy in the shortest time possible.

In the joint reconstruction technique, following the resection of the ankylosis block, the structure that is compromised is restored to establish the vertical height and the condylar structure, aiming to improve function. Autogenous grafts – such as costochondral, iliac crest or cononoid process grafts – or alloplastic materials – such as articular prostheses – may be used.^5^^,^^13^ Costochondral grafts are the most widely accepted; they are biologically compatible and functionally adaptable.^14^ The growth potential of this type of graft makes it the material of choice in children.^13^^,^^15^ Problems with costochondral grafts include fractures, reankylosis, donor site morbidity, and variable graft growth.^14^ This technique is indicated for bilateral osseous ankylosis cases where there is intra- or extra-articular involvement. Coronoid process grafts make it possible to reconstruct the condyle through the preauricular approach, avoiding donor site damage, as the coronoid process is used in loco.^16^ This technique yields good results in adult patients that have major ankylosis. This type of graft was chosen for the adult patients in this study (n=3), mainly as it does not require another donor site, thus reducing the morbidity. The costochondral graft was used in one pediatric patient (no. 1).

A number of alloplastic materials have been developed to avoid these problems, such as acrylic resin, synthetic fibers and full titanium joints. Alloplastic joints make it possible to reproduce more closely the natural joint anatomy, restoring the vertical height, decreasing surgery time, and reducing the rate of recurrences.^8^ Borçbakan apud Ko et al.^15^ first used an acrylic condyle to treat TMJ ankylosis. Acrylic is a simple, inexpensive, and easily manufactured material; it does not require another donor site and is well-tolerated in the body.^8^ The only disadvantage of this technique is that facial asymmetry may develop when used in children.^8^ Our sample patients were no longer in any growing phase and their disease was fairly recent; the coronoid process, therefore, could not be used, given its small size. Graft material, then, were acrylic resin prostheses, made by a buccomaxillofacial prosthetist.

Regardless of the technique chosen, surgeons should undertake aggressive resection of the fibrosed or osseous ankylotic segment to avoid recurrences. Additionally, dissection of the mandibular ramus muscles and ipsilateral coronoidectomy should be done to avoid intraoperatory mouth opening limitations, as the coronoid process may be elongated in cases of ankylosis of long duration.^17^ If a 35mm passive mouth opening is not attained, contralateral coronoidectomy should be done. Physical therapy is recommended following these procedures to prevent and to undo adhesions, to avoid soft tissue contraction, and to foster normal muscle function.^17^^,^^18^ Some authors prefer to wait 5 to 7 days for pain and edema to subside and initial healing of soft tissues to take place before implementing physical therapy, as early mandible mobilization may cause bleeding and hematomas, which would delay healing.^18^ Drainage tubes stop blood from accumulating in the newly formed cavity and facilitate postoperative movements.

Facial nerve injury may occur if there is excessive intraoperatory retraction of tissues.^2^^,^^19^ The prevalence of facial nerve injury varies from 9% to 18%.^19^ Such damage is minimized when the surgical team is experienced, and when the surgical approach is adequately chosen. Possibilities include Al Kayat and Bramley's apud Roychoudhry^2^ modified preauricular incision and Ellis and Zide's preauricular incision.^10^ The latter incision was used in this study; it was effective in avoiding facial nerve injury in all of the sample patients.

The most frequent complications of surgery for the treatment of ankylosis are: limited mouth opening, reankylosis, and occlusal defects.^3^ Careful surgical technique and meticulous physical therapy of long duration are essential to avoid complications and attain satisfactory results.^6^ Only one patient (no. 1) in this study, in whom the autogenous graft reconstruction technique was used, had limited maximum mouth opening after surgery (preop – 0mm; postop – 14mm). This was the only case that relapsed.

## CONCLUSION

Reconstruction of the joint using autogenous or allogenic materials for the treatment of TMJ ankylosis is effective, considering the postoperative maximum mouth opening, recurrence and function of the joint.
